# *Clostridioides difficile* in Pigs and Dairy Cattle in Northern Italy: Prevalence, Characterization and Comparison between Animal and Human Strains

**DOI:** 10.3390/microorganisms11071738

**Published:** 2023-07-02

**Authors:** Patrizia Spigaglia, Fabrizio Barbanti, Silvia Faccini, Mariella Vescovi, Enrico Maria Criscuolo, Rossella Ceruti, Clara Gaspano, Carlo Rosignoli

**Affiliations:** 1 Dipartimento di Malattie Infettive, Istituto Superiore di Sanità, 00161 Roma, Italy; 2 Istituto Zooprofilattico Sperimentale della Lombardia e dell’Emilia Romagna “B. Ubertini”, Sede Territoriale di Mantova, 46100 Mantova, Italy; 3 Servizio di Medicina di Laboratorio, ASST Ospedale “Carlo Poma”, 46100 Mantova, Italy

**Keywords:** *Clostridioides difficile*, CDI, one health, animal, human, food, PCR-ribotyping, antibiotic resistance

## Abstract

It has been observed that novel strains of *Clostridioides difficile* can rapidly emerge and move between animal and human hosts. The aim of this study was to investigate the prevalence of *C. difficile* in pigs and dairy cattle in northern Italy and to characterize and compare *C. difficile* animal strains with those from patients from the same geographical area. The *C. difficile* strains were isolated from animals from farms and slaughterhouses (cross-sectional studies) and from neonatal animals with enteric disorders in routine diagnostic investigations (passive surveillance). Samples positive for *C. difficile* were found in 87% of the pig farms and in 40% of the cattle farms involved in the cross-sectional studies, with a 20% prevalence among suckling piglets and 6.7% prevalence in neonatal calves, with no significant difference between animals with and without diarrheal symptoms. The prevalence of *C. difficile* in older animal categories was significantly lower. This result suggests that young age is an important risk factor for *C. difficile* colonization. In cross-sectional studies at slaughterhouses, in both the heavy pigs and dairy cows examined, only 2% of the intestinal content samples were positive for *C. difficile* and no contamination was found on the surface of the carcasses. Considering passive surveillance, the prevalence rates of positive samples were 29% in piglets and 1.4% in calves. Overall, 267 strains of animal origin and 97 from humans were collected. In total, 39 ribotypes (RTs) were identified, with RT 078 and RT 018 being predominant among animals and humans, respectively. Several RTs overlapped between animals and patients. In particular, RT 569 was identified as an emergent type in our country. Resistance to erythromycin and moxifloxacin was widely diffused among *C. difficile* strains, regardless of origin. This study supports *C. difficile* as a pathogen of one-health importance and highlights the need for a collaborative approach between physicians and veterinarians to control and prevent infections that are able to cross species and geographical barriers.

## 1. Introduction

*Clostridioides difficile* is an anaerobic, toxin-producing, antimicrobial resistant bacterium, known as the main cause of diarrhea and pseudomembranous colitis in elderly and hospitalized patients treated with antibiotics [1]. Dramatic increases in the incidence and severity of *C. difficile* infection (CDI), as well as in the associated morbidity and mortality, have recently been observed not only in hospitals but also in the community [2,3,4,5].

Changes in *C. difficile* epidemiology have been associated with the emergence of highly virulent types, such as PCR ribotype (RT) 027, responsible for many large-scale outbreaks and deaths worldwide in recent decades [6,7,8]. Several emergent RTs are recognized as the causes of both hospital-acquired infections (HA-CDI) and community-acquired infections (CA-CDI). In particular, strain RT 078 has been identified as a common cause of CA-CDI in humans but also in animals, particularly in food animals and household pets [9,10,11]. The risk factors associated with CA-CDI have not been clearly identified. In fact, CA-CDI patients are usually younger compared to HA-CDI patients, and the use of antibiotics has been identified as a risk factor for CA-CDI in some studies, whereas other authors report that CA-CDI patients are significantly less or not exposed to antibiotics compared with HA-CDI patients [12,13]. Furthermore, strains isolated from CDI cases in the community have been found to be more heterogeneous compared to those from hospitals, often including strains belonging to previously unidentified RTs [14,15]. These observations suggest that *C. difficile* can be acquired outside of the hospital settings. In fact, *C. difficile* is established not only in the healthcare system but also in a range of ecological niches, including the environment and many animal species [16,17]. In particular, animals may represent an important reservoir of *C. difficile* for human CA-CDI [18]. In particular, the potential zoonotic transmission of this pathogen is supported by the overlap of *C. difficile* RTs between humans and animals and the recent findings showing colonization of pigs and farmers by the same clonal RT 078 isolates [19,20,21,22,23,24]. New *C. difficile* ribotypes may rapidly emerge and spread through the global health care system, as demonstrated for the RT 027 lineage [25], and also move between animal and human hosts, with no geographical barriers, as demonstrated for the RT 078 lineage [26,27,28]. In particular, *C. difficile* has frequently been detected in both healthy and symptomatic food animals [11,29,30,31]. In addition, the detection of *C. difficile* in retail meat and the resistance of *C. difficile* spores to temperature has raised concerns about the possibility that the consumption of raw contaminated foods could lead to colonization and infection by *C. difficile* in humans [32,33,34,35,36,37,38].

An increasing number of studies indicates that resistance to antibiotics plays an important role in driving CDI epidemiological changes. In fact, it has been demonstrated that the spread of highly virulent RTs is correlated with the acquisition of resistance to antibiotics (resistance to fluoroquinolones for *C. difficile* RT 027, to tetracycline for RT 078 and to clindamycin for RT 017) [25,26,39,40,41]. *C. difficile* transfer between humans and animals, as observed for *C. difficile* strain RT 078 [21,22,42], may represent an important and under-estimated route of antibiotic resistance gene dissemination. The rapid acquisition and diffusion of antibiotics resistance among the *C. difficile* population are highlighted by the emergence and spread among the healthcare systems of *C. difficile* RTs lineages showing an extensive repertoire of antibiotic resistance determinants [25,26,39,43,44]

In this collaborative study, funded by the Italian Ministry of Health, we investigated a large number of samples collected from March 2017 to May 2019 from food animals (pigs and dairy cattle) and human patients living in northern Italy. The study was carried out with the following aims: (i) to investigate the prevalence of *C. difficile* in fecal samples of both healthy and diarrheic pigs and dairy cattle directly on farms (cross-sectional studies at farms); (ii) to investigate the prevalence of *C. difficile* in intestinal contents and carcasses of heavy fattening pigs and retired dairy cows at slaughterhouses (cross-sectional studies at slaughterhouses); (iii) to investigate the prevalence of *C. difficile* in samples from diarrheic neonatal piglets and calves (passive surveillance); (iv) to characterize and compare *C. difficile* animal strains and strains from patients with CDI in the same geographical area.

## 2. Materials and Methods

### 2.1. Collection of Animal Samples

**Figure 1:**
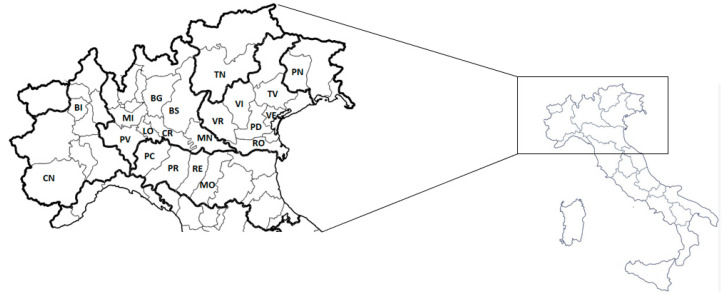
The provinces of northern Italy involved in this study. Mantova (MN), Cremona (CR); Brescia (BS), Pavia (PV), Milano (MI) and Bergamo (BG) are located in the Lombardia region; Modena (MO), Reggio Emilia (RE), Piacenza (PC) and Parma (PR) in the Emilia-Romagna region; Verona (VR), Vicenza (VI), Padova (PD), Rovigo (RO, Treviso (TV) and Venezia (VE) in the Veneto region; Trento (TN) in the Trentino-Alto Adige region; Pordenone (PN) in the Friuli Venezia Giulia region; and Biella (BI) and Cuneo (CN) in the Piemonte region.

The animal samples analyzed in this study were collected between March 2017 and May 2019 from different provinces of northern Italy (Figure 1). Some of the animal samples from pigs and calves were actively collected by the Mantova laboratory of the “Istituto Zooprofilattico Sperimentale della Lombardia e dell’Emilia Romagna” (IZSLER), in collaboration with farms and slaughterhouse veterinarians (cross-sectional studies); the remainder were submitted to the IZSLER for diagnostic investigations in neonatal animals with diarrhea (passive surveillance).

In total, 2139 animal samples were collected from different provinces in northern Italy. Overall, 1439 samples were obtained from pigs and 700 from dairy cattle. In particular, the cross-sectional studies included 1080 samples from heathy and diarrheic animals from farms (720 from swine and 360 from cattle), and 426 samples from animals from slaughterhouses, 226 from pigs (113 carcass swabs and 113 intestinal content samples) and 200 from dairy cows (100 carcass swabs and 100 intestinal content samples). The passive surveillance included 633 samples collected from neonatal animals with diarrhea (140 from calves and 493 from piglets).

#### 2.1.1. Collection of Porcine Samples

In the cross-sectional study on the pig farms, fecal samples from suckling piglets were collected from healthy and diarrheic live animals; 450 fecal samples were collected from 15 different farrow-to-finish swine farms (30 samples from each farm) located in six different northern Italian provinces. In addition, 270 fecal samples from healthy older pigs were collected from the three pig farms with the highest prevalence of *C. difficile* isolation in neonatal piglets. Farms were included in the study if the herd owner and their veterinary consultant were willing to give their sampling support.

In the cross-sectional study on slaughter pigs, sampling of the intestinal contents and carcass swabs from heavy fattening pigs (160–180 Kg b.w.) was carried out in a large slaughterhouse in the province of Mantova, with a daily slaughtering activity rate of about 3000 pigs. The samples were taken on 6 different days in April 2019 and were collected from 113 animals from 75 farms located in 16 different provinces of northern Italy. Every pig included in the study was randomly selected from a batch of 100–140 pigs, all coming from the same farm.

In the passive surveillance study on neonatal piglets with diarrhea, samples were sent to the IZSLER laboratory for routine diagnostic investigations. Fecal samples from living animals or large intestinal contents from carcasses with gross intestinal lesions referable to enterocolitis at the post-mortem examination were selected for this study. In total, 137 diarrheic samples and 356 carcasses of sucking piglets (first 2 weeks of life) were collected from 52 farms located in 13 different provinces of northern Italy.

#### 2.1.2. Collection of Bovine Samples

In the cross-sectional study in cattle farms, 150 fecal samples from both healthy and diarrheic neonatal calves were collected in 15 different dairy farms (10 samples from each farm), all located in the province of Mantova. In addition, 210 fecal samples from cattle of all ages were collected from the three dairy cattle farms with the highest prevalence of *C. difficile* isolation in neonatal calves.

In the cross-sectional study on slaughter cattle, sampling of the intestinal contents and carcass swabs was performed on five different days in the months of April and May 2019 in a slaughterhouse located in the province of Mantova. One hundred adult cattle at the end of the production cycle from specialized dairy farms were subjected to investigation. The 100 cows sampled came from 89 different farms located in 13 different provinces in northern Italy.

In the passive surveillance study on neonatal calves with diarrhea, 36 diarrheic samples and 104 intestinal content from carcasses of newborn calves (first 3 weeks of life) were collected. The fecal samples and carcasses originated from 98 farms located in 7 different provinces of northern Italy.

### 2.2. Collection of Human Strains

Human *C. difficile* strains, isolated from consecutive diarrheic patients with suspected CDI between March 2017 and February 2018 in the microbiological laboratory of the “Carlo Poma” hospital of Mantova, were included in the study. Only one strain from each patient was included in the study. The human samples analyzed came from the hospital, the long-term care facilities and the community of the province of Mantova (MN), which includes the majority of pig and cattle farms involved in the study.

### 2.3. C. difficile Sampling and Storage

Fecal rectal samples from neonatal animals were collected using a sterile swab, while fecal rectal samples from older animals were collected using a gloved hand. Stool samples were immediately stored at 4 °C and sent to the IZSLER laboratory within 24 h.

Sampling in the slaughterhouses was carried out during the post-evisceration phase and before chilling. A portion of the cecum content was collected from each animal enrolled. The sampling was carried out on four different areas (each of about 100 cm^2^) of the carcass’ surface using a single hydrated sponge with 10 mL of buffered peptone water, as indicated by the ISO 17604:2015 procedures [45].

Fecal samples, intestinal content samples and carcass sponges were stored at 4 ± 3 °C and analyzed for the presence of *C. difficile* within 24–48 h of collection.

### 2.4. C. difficile Isolation

In the passive surveillance studies, *C. difficile* isolation from animal feces or intestinal contents was performed after ethanolic shock to induce endospore germination. The samples were mixed with 95% ethanol in a 1:1 (*v*/*v*) ratio and left for 30 min at room temperature. Then, the mixture was inoculated onto selective *C. difficile* taurocholate cycloserine cefoxitin fructose agar plates (TCCFA), a *Clostridium difficile* agar base and a *Clostridium-difficile*-selective supplement (Oxoid Limited, Basingstoke, UK) supplemented with 5% defibrinated sheep blood, and the plates were incubated in a jar with an anaerobic atmosphere generation system (Oxoid Limited, Basingstoke, UK) at 37 °C for 48 h.

In the cross-sectional studies, for *C. difficile* isolation from feces, intestinal contents or carcass swabs, an enrichment step was performed before the ethanolic shock, consisting of anaerobic incubation for 7 days of 1 g of feces/intestinal content or a carcass swab in 9 mL or 40 mL, respectively, of taurocholate cycloserine cefoxitin fructose broth (TCCFB).

The *C. difficile* strains from human samples of diarrheic patients with suspected CDI were isolated on selective chromID™ *C. difficile* plates (bioMérieux, Marcy l’Etoile, France) after 48 h of incubation in an anaerobic cabinet (90% N_2_, 5% CO_2_ and 5% H_2_). After growth on selective plates, the isolated single colonies of *C. difficile* were then inoculated onto blood agar plates (BA) supplemented with 5% sheep blood, 5 mg/L haemin and 0.5 mg/L vitamin K, then after 24 h of incubation in an anaerobic atmosphere the cultures were stored in cryotubes at −80 °C for subsequent analysis.

### 2.5. DNA Extraction and C. difficile Identification

Bacterial DNA extraction was performed by suspending several *C. difficile* fresh colonies in 100 μL of 5% *w*/*v* Chelex-100 resin (Bio-Rad, Hertfordshire, UK) in molecular-grade H_2_O. The bacterial suspensions were heated to 100 °C for 10 min and the lysates were centrifuged for 3 min at 16,000× *g*. The supernatants were collected and the DNA concentration was adjusted to 100 ng/μL.

The isolates were identified as *C. difficile* if the presence of the triose phosphate isomerase (*tpi*) gene was confirmed by PCR [46].

### 2.6. C. difficile Molecular Toxin Profile and Typing

In total, 267 *C. difficile* strains from animals (250 from pigs and 17 from calves) and 97 strains from humans were characterized in this study.

A multiplex PCR was performed to test the presence of the genes coding for toxins A and B (*tcdA* and *tcdB*) and the genes coding for the binary toxin (*cdtA* and *cdtB*), as suggested by the European Centre for Disease Prevention and Control (ECDC) [47]. The PCR assay also included two controls to test for appropriate DNA isolation and *C. difficile* identification, respectively.

The *C. difficile* typing was performed using the capillary PCR ribotyping method and the free web database WEBRIBO (http://webribo.ages.at) (accessed on 1 March 2023), as previously described [48].

The different patterns of peaks generated by the capillary PCR ribotyping were compared using GelComparII v 6.6 (Applied Maths, Sint-Martens-Latem, Belgium) and analyzed for similarity with the Dice coefficient, with 2% optimization. The clustering was performed using the unweighted pair group mean association (UPGMA) method and the *C. difficile* isolates were considered closely related if they showed a percentage of similarity ≥ 80%.

### 2.7. Molecular Analysis of Resistance Mechanisms

The detection of the *ermB* gene was performed by amplifying an internal fragment of the gene using the primer pair E5/E6 [49], whereas primer pairs described by Patterson et al. [50] were used to detect the presence of other classes of *erm* genes (*C*, *F*, *G* and *Q*).

The primer pair TETMd/TETMr was used to detect the presence of the *tetM* gene [51], whereas other *tet* classes (*O*, *Q* and *W*) were investigated using a specific set of primers that had already been published [50].

Mutations in the *gyrA* and *gyrB* genes in *C. difficile* strains resistant to fluoroquinolones were detected as previously described [52]. Briefly, the quinolone resistance-determining region (QRDR) of both *gyrA* and *gyrB* genes was amplified using two different couples of primers, and subsequently the PCR product was sequenced and analyzed for mutations using Geneious 9.1.8 (Biomatters Ltd., Auckland, New Zealand).

### 2.8. Antimicrobial Susceptibility Testing

The minimum inhibitory concentrations (MICs) for moxifloxacin (MXF), erythromycin (ERY), tetracycline (TET), amoxicillin (AMX), metronidazole (MTZ) and vancomycin (VAN) were evaluated by E-test (bioMérieux, Marcy l’Etoile, France) onto pre-reduced BA plates supplemented with 5 mg/L hemin, 1 mg/L vitamin K1 (Sigma Aldrich, Darmstadt, Germany) and 5% defibrinated sheep red blood cells. The MIC values were recorded after 48 h of incubation in anaerobic conditions.

The breakpoint used for ERY and MXF was 8 mg/L, while the breakpoint for TET and AMX was 16 mg/L, in accordance with the CLSI interpretative categories approved for anaerobic bacteria [53]. The resistance to metronidazole MTZ and VAN was defined as MIC > 2 mg/L, according to the epidemiological cut-off values (ECOFFs) suggested by the European Committee on Antimicrobial Susceptibility Testing [54].

The Wilkins–Chalgren-based agar incorporation method was used as previously described [55] to re-evaluate strains showing MICs for VAN > 2 mg/L by E-test.

### 2.9. Statistical Analysis

A two-tailed Fisher exact test was used to assess the associations between categorical variables, and a *p* value < 0.05 was considered statistically significant. The analyses were carried out using GraphPad Prism 5 software (GraphPad Software, San Diego, CA, USA).

## 3. Results

### 3.1. Sampling and C. difficile Isolation from Animals and Humans

In total, 267 (12.5%) animal samples were found to be *C. difficile*-positive (Table 1). In the cross-sectional study on the farms, 20% (90/450) of the samples collected from suckling piglets were positive for the presence of *C. difficile* (Table 1).

There was no significant difference (*p* > 0.05) between the number of samples positive for *C. difficile* in healthy (51/271, 18.8%) and diarrheic suckling piglets (39/179, 21.8%). Overall, 86.7% (13/15) of the farms involved showed at least one sample from a neonatal piglet positive for *C. difficile*. A total of 5.6% (15/270) of the fecal samples from healthy pigs of the oldest ages were found to be positive for *C. difficile* (two strains from weaned pigs, one strain from a finisher pig and 12 strains from breeding sows). These pigs of the oldest ages were from the three farrow-to-finish pig farms in which the highest prevalence of *C. difficile* isolation from suckling piglets was observed.

In general, in the cross-sectional study on the cattle farms, 6.7% (10/150) of the samples collected from neonatal animals were positive for the presence of *C. difficile* (Table 1). As well as for suckling piglets, no significant differences (*p* > 0.05) were observed between samples positive for *C. difficile* in healthy (7/99, 7.1%) and diarrheic neonatal calves (3/51, 5.9%). In 40% (6/15) of the cattle farms involved in the study, at least one sample from the neonatal calves was positive for *C. difficile* (Table 1). In the three farms with the highest prevalence of *C. difficile* isolation in neonatal calves, 1.4% (3/210) of the fecal samples from healthy cattle of the oldest ages were found to be positive for *C. difficile*: one from a 4–5-month-old heifer, one from a 6–12-month-old heifer and one from a 19–24-month-old heifer.

Finally, samples from 113 pigs and 100 dairy cows at slaughterhouses were included in this cross-sectional study, and only two pigs (1.8%) and two cows (2.0%) were found to be intestinal carriers of *C. difficile*. No swabs from carcasses were positive for *C. difficile* (Table 1).

During the passive surveillance on neonatal animals with diarrhea, 332 samples of the 633 investigated (52%) were collected from farms located in the Mantova area. *C. difficile* was detected in 22.9% (145/633) of these samples, mostly collected from swine (143). In 60% (31/52) of the pig farms participating in this study, at least one sample positive for *C. difficile* was found. The two isolates from neonatal calves were collected in two different farms of the 98 dairy cattle farms involved, one from the intestinal content of a dead animal (1/104) and one from the diarrheic feces of a living animal (1/36). Interestingly, moderate to severe mesocolonic edema was observed in 24% (85/356) of the piglet carcasses examined. A significant difference in the percentages of the intestinal content samples positive for *C. difficile* (*p* < 0.01) was observed between piglets with and without this macroscopic intestinal lesion, at 69.4% (59/85) and 19.6% (53/271), respectively.

In total, 97 *C. difficile* isolates from humans (64 female and 33 male) were included in the study, of which 61 were from the hospital, 25 from the community and 11 from long-term care facility (LTCFs) patients, with an average age of 78 years (Table 2).

### 3.2. C. difficile Molecular Toxin Profile and Typing

Four different profiles were identified. The majority of animal strains (94%) showed a *tcdA*+/*tcdB*+/*cdtA*+/*cdtB*+ profile, while 81% (79/97) of the human strains showed a *tcdA*+/*tcdB*+/*cdtA*−/*cdtB*− profile (Table 3). Interestingly, 2.6% (7/267) of the animal strains were *tcdA*+/*tcdB*−/*cdtA*+/*cdtB*+, while strains with this profile were not detected among the human strains. Four strains, two from animals and two from humans, were negative for toxin genes.

In total, 11 different RTs were identified among the animal strains and 28 RTs among the human strains (Table 1 and Table 2). In general, RT 078 was found to be prevalent among animals (84%), while the majority of human strains (39%) belonged to RT 018.

Nine RTs (RT 023, RT 045, RT 033/1, RT 066/2, RT 078, RT 126, RT 427, RT 620 and RT 743) were positive for the toxin A, toxin B and CDT genes. The other RTs identified in this study were positive for the toxin A and toxin B genes, except for strain RT 033, which was positive for the toxin A and CDT genes, and strains RT 085 and PR 23597, which were non-toxigenic.

**Figure 2:**
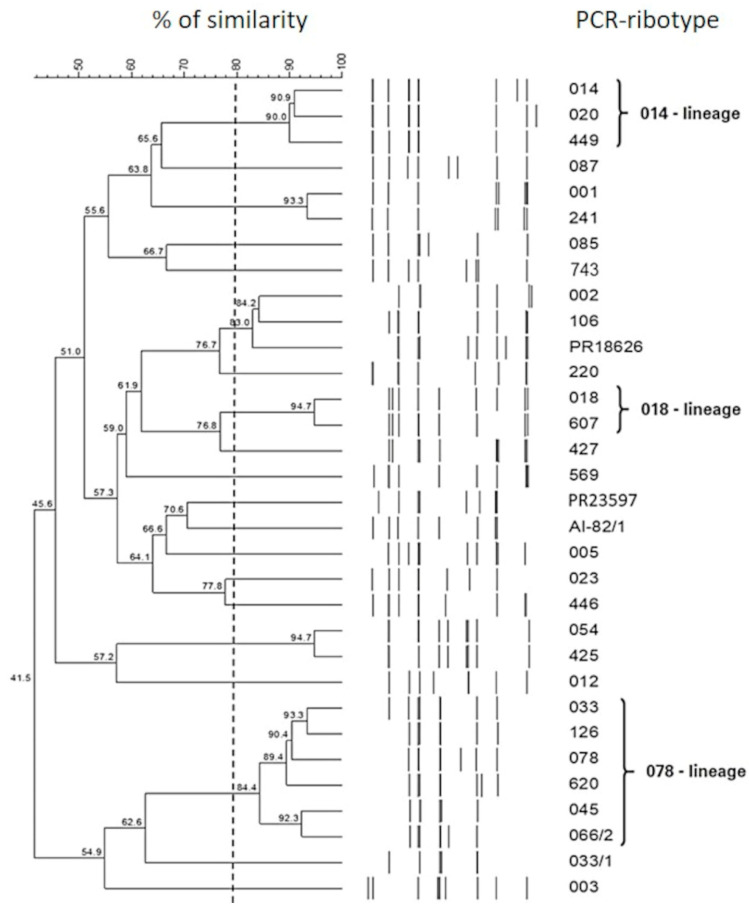
Phylogenetic tree obtained by PCR ribotyping the *C. difficile* strains investigated in this study. A similarity analysis was performed with Dice’s coefficient along with clustering using the unweighted pair group mean association (UPGMA) method.

Eight different RTs were detected among the porcine strains and five RTs among the bovine strains (Table 1). In particular, RT 078 with RT 033, RT 045, RT 066/2, RT 126 and RT 620, all belonging to the RT 078 lineage, included 97% (259/267) of the *C. difficile* animal strains analyzed in this study (Figure 2). Interestingly, 41% (7/17) of the bovine strains belonged to RT 033, a type that was not detected among the swine. In general, strains of RT 078 were isolated from both swine and calves, either symptomatic or not, located in almost all the sampled farms.

Interestingly, strains belonging to an emerging type in Italy, RT 569, were isolated only in pigs from two farms located in different provinces.

Overall, 42% (41/97) of the human strains were grouped in the RT 018 lineage that included RT 018 and RT 607, while 11% (11/97) were recognized as RT 014, RT 020 or RT 449, all belonging to the RT 014 lineage (Table 2 and Figure 2). The ribotypes RT 002, RT 005, RT 014, RT 018, RT 220, and RT 607 were detected in both patients with CA-CDI and HA-CDI, while RT 569 was only from CA-CDI.

Seven different RTs (RT 001, RT 005, RT 078, RT 085, RT 126, RT 569 and RT 620) were detected in both animals and humans (Table 4), while RTs belonging to both the RT 018 lineage and RT 014 lineage were not found in animals. Among the RTs found in both humans and animals, RT 569 was detected only from CA-CDI, while RT 005 was detected from both CA-CDI and HA-CDI and the other RTs were only detected from HA-CDI.

### 3.3. Antibiotic Susceptibility

A selection of *C. difficile* strains, 155 from animals (140 porcine and 15 bovine) and 95 from humans, were investigated for antibiotic susceptibility using the E-test method. In total, 141 (91%) animal strains and 64 (67%) human strains were resistant to at least one of the antibiotics tested (Table 5). High percentages of both animal (88%) and human (62%) strains were resistant to ERY. Forty eight percent of both animal and human strains were resistant to MXF, while resistance to TET was observed only in one human strain. Resistance to MTZ, VAN and AMX was not observed in either animals or humans, although seven human strains showed an MIC of 2 mg/L for VAN.

The majority of animal strains belonging to the RT 078 lineage (RT 033, RT 045, RT 066/2, RT 078, RT 126 and RT 620) were resistant to at least one of the antibiotics tested (Table 5). Among the strains of this lineage, only those belonging to RT 033 were susceptible to all antibiotics tested. The majority of human strains analyzed were resistant to at least one of the antibiotics tested in the study, specifically all strains of RT 078, 98% of the strains belonging to the RT 018 lineage and 20% of the strains grouped in the RT 014 lineage (Table 5). Interestingly, 27/32 strains from healthy animals and 115/125 strains from symptomatic animals were resistant to one or two classes of antibiotics. *C. difficile* strains resistant to antibiotics were isolated from most of the farms with animals positive for this pathogen (89.3% 50/56).

### 3.4. Antibiotic Resistance Mechanisms

An analysis for resistance mechanisms was performed on 202 *C. difficile* strains (133 from suckling piglets, seven from neonatal calves, one from a 3–5-month-old heifer and 61 from hospital patients). All strains with intermediate MIC values for TET (corresponding to E-test values of 4, 6, 8 and 12 mg/L) were also investigated for mechanisms of resistance because it is known that strains with intermediate MICs can show an inducible resistance in the presence of sub-inhibitory concentrations of TET [49].

In general, considering the detection of genes or mutations involved in antibiotic resistance, 17 different profiles were detected in pigs and six profiles in cattle (Table 6). Among the 137 animal strains resistant to ERY, 24% (33/137) were positive for an *erm* gene. In particular, the *ermB* gene was the most commonly found (32/137), while *ermQ* was found only in one strain. Among the 64 animal strains with intermediate MICs for TET, 59 were positive for *tetM.* Among the strains showing a *tetM* gene, 37 strains also contained a *tetO* gene, nine both a *tetO* and a *tetW* gene and two a *tetW* gene (Table 6). Only one animal strain was positive for only *tetO*. Among the 74 animal strains resistant to MXF, 73 showed the amino acid substitution Thr82Ile in GyrA and one porcine strain showed the substitution Thr82Val. Animal strains with several genes and mutations conferring resistance to antibiotics were more frequently isolated in two farms of the province of Brescia, two farms of the province of Mantova and one farm of the province of Modena. In particular, 46% of animal strains of the RT 078 lineage showed two or more antibiotic resistance mechanisms.

Nine different molecular profiles were identified in human strains when considering the detection of genes or mutations implicated in antibiotic resistance (Table 7). Among the 59 human strains resistant to ERY, 22% (13/59) were positive for the *ermB* gene, three strains were positive for *ermQ* and one was positive for *ermC*. All of the 46 human strains resistant to MXF showed a substitution of Thr82Ile in GyrA. Interestingly, only 19% of human strains belonging to the RT 018 lineage showed more than one antibiotic resistance mechanism, while the percentage was 60% among human strains of the RT 078 lineage (Table 7).

## 4. Discussion

This study provides data on the CDI prevalence in pigs and dairy cattle from northern Italy and an accurate characterization and comparison of a large number of both animal and human *C. difficile* isolates from this geographic area.

Data obtained from neonatal living animals from farms showed that at least one sample positive for *C. difficile* was found in 87% of the pig farms and 40% of the dairy cattle farms included in the study. In total, considering the cross-sectional studies on the farms, 10.9% of the animal samples were positive for *C. difficile*, with a higher prevalence in swine (14.6%, 105/720) compared to cattle (3.6%, 13/360). This result is not surprising, since *C. difficile* is a well-known pathogen for pigs, in particular for neonatal piglets [31,56,57,58]. In fact, the rate of mortality associated with CDI can reach 50% in suckling piglets, with as many as 58% of the surviving animals showing weight loss [31,59]. In our study, a significant percentage (69.4%) of positive piglet carcasses presented mesocolonic edema, a characteristic lesion already described by other authors in CDI cases [31,59,60,61]. Although it cannot be considered pathognomonic, our data suggest a significant association between mesocolonic edema and symptomatic CDIs in piglets.

The *C. difficile* prevalence rate found in suckling piglets in the study at farms (20%) was lower than those reported in other countries, ranging from 27.7% in the Czech Republic and 73% in Germany [62,63,64,65,66,67,68]. The heterogeneity of *C. difficile* prevalence values observed may likely be affected by several factors, such as geographical and environmental characteristics, the animal breed, the antibiotic treatment and the rearing method. The prevalence of *C. difficile* in cattle also varies widely from one study to another, with percentages ranging between 0% and 60% [69,70,71,72,73]. In the present study, calves positive for *C. difficile* were found in different farms located in the area of Mantova, and the majority of them were neonatal animals (70%). A higher prevalence of *C. difficile* in neonatal calves and piglets compared to adult animals is frequently described, probably due to less developed gut microbiota that may facilitate *C. difficile* colonization and proliferation and the production of toxins in younger animals [74].

An important finding that emerged from our survey on animals on farms is that *C. difficile* was isolated from both symptomatic and healthy animals, without significant differences between the number of positive samples for healthy or diarrheic neonatal animals (21.8% in symptomatic animals vs. 18.8% in asymptomatic animals). Interestingly, only 6% (15/270) of samples from older pigs were positive for *C. difficile*, showing that piglets are the main carriers of this pathogen, which is probably acquired from the surrounding contaminated environment rather than vertical transmission [30,61]. *C. difficile* pathogenesis in piglets seems complex, and it is probably affected by several factors other than the underdevelopment of intestinal microflora [75,76]. In this study, an association between the presence of *C. difficile* toxigenic strains and a symptomatic status of piglets was not found, since toxigenic strains were equally detected in healthy and sick animals, highlighting the importance of asymptomatic carriers as reservoirs of this pathogen.

Heterogeneous prevalence values in the intestinal contents of food animals at slaughter have been reported in the literature [77,78,79,80,81]. In particular, a high *C. difficile* prevalence (25.3%) was observed in neonatal calf carcasses at slaughter in Australia [80]. As hypothesized by the authors, the younger age of the animals analyzed could partially explain the high prevalence of *C. difficile* observed, since calves in Australia are slaughtered 7–14 days after birth, while in North America and Europe, they are slaughtered at between 21 and 27 weeks of age [79,82]. Although our data indicate that a low number of dairy cattle and finisher pigs harbored *C. difficile* when they entered the food chain (1.8% of heavy pigs and 2.0% of dairy cows), these animals could represent a source of toxigenic *C. difficile* contamination of meat processing facilities at the time of harvest. For this reason, the careful application of hygienic measures in slaughterhouses should be systematically ensured to avoid the spillage of digestive tract contents during and after evisceration.

In addition to RT 078, which is known to be widely diffused in both humans and animals [22,83], our data showed that other RTs (RT 001, RT 005, RT 085, RT 126, RT 620 and RT 569) overlapped between animals and humans. In particular, RT 569 was not only isolated from pigs (consistent with our previous findings [84]) but also from CA-CDI patients. This observation suggests the possible circulation of strain RT 569 between animals and humans in the community, although this hypothesis needs to be confirmed by further phylogenetic analysis. RT 033 (41%) and RT 078 (23%) were the most common RTs detected in both symptomatic and healthy calves, in accordance with previous studies [85,86,87,88]. Although positive for toxin A and the CDT genes, *C. difficile* strain RT 033 only produced CDT, due to a large deletion in the pathogenicity locus (PaLoc) [89]. Nevertheless, RT 033 is able to cause infection not only in animals but also in humans [90,91], with a higher risk of false diagnosis when enzymatic assays for toxin A and B are used [84,92,93].

The susceptibility analysis showed that the animal and human strains investigated in this study had high percentages of resistance to ERY and MXF, with the majority of both animal and human strains being found to be negative for the presence of *erm* genes. Resistance in *erm*-negative strains may be due to other accessory genes conferring resistance to MLSB antibiotics. In particular, the *cfr* genes, encoding a 23S rRNA methyltransferase, and the *cme* gene, encoding for a multidrug transporter, have been found to be implicated in resistance to MLSB antibiotics [94,95].

The percentage of *C. difficile* strains from cattle resistant to MXF (13%) was similar to the values recently reported in other studies [86,87]. Conversely, the percentage of porcine strains resistant to MXF (52%) was higher compared to the values reported by other authors [65,96,97,98], and also in comparison with the percentage found in human strains (48%) in this study. Resistance to MXF is usually conferred by the amino acid of substitution Thr82Ile in GyrA in the majority of *C. difficile* strains resistant to fluoroquinolones [99]. Despite a recent reduction in the consumption of fluoroquinolones in veterinary and human medicine [100,101], this class of antibiotics is still highly used in Italy, and this fact could partially explain the high prevalence of *C.-difficile*-resistant strains in our country [84].

Considering the detection of genes or mutations involved in antibiotic resistance, a higher heterogeneity in antibiotic resistance molecular profiles was observed in animal strains compared to human strains. Interestingly, although all *C. difficile* animal strains were susceptible to TET in this study, the molecular analysis showed that these strains contained one or more *tet* genes. It has previously been observed that susceptible *C. difficile* isolates that are *tet*-positive show an inducible resistance to TET when subjected to sub-inhibitory concentrations of this antibiotic [49]. *C. difficile* strains with inducible resistance to TET may be clinically relevant for animals in consideration of the wide use of this class of antibiotics in veterinary medicine [40]. In addition, the widespread use of tetracycline appears to be driving the expansion of *C. difficile* clones resistant to this antibiotic, particularly in RT078 [21,26,40].

All the *C. difficile* animal strains analyzed in this study showed full susceptibility to AMX, MTZ and VAN. While in human medicine, the use of penicillins is reported to be frequently associated with CDI [102], only a few studies on horses and calves have reported increased intestinal exposure to *C. difficile* associated with the administration of these antibiotics [79,103]. MTZ and VAN are considered the first-line treatments for non-severe CDI and severe CDI, respectively, in humans. Although the percentage of *C. difficile* strains resistant to MTZ and VAN is still low, an increasing number of studies reports *C. difficile* strains with reduced susceptibility or resistant to these antibiotics in both humans and animals [99].

Some general considerations emerged from this study. The first one is that *C. difficile* is fairly widespread in pigs, and to a lesser extent, in cattle farms in northern Italy. *C. difficile* is predominantly isolated from neonatal animals in both pigs and dairy cattle, showing that a young age is an important risk factor for CDI in animals [11].

Although the prevalence of toxigenic *C. difficile* samples found in the intestinal contents of pigs and cattle at slaughter was low, our results suggest a potential risk of contamination of retail meat destined for human consumption. In fact, despite *C. difficile* being unable to grow in foods due to the absence of bile salts and the fact that there is no current epidemiologic evidence supporting that it is a food-borne pathogen, this bacterium can survive the cooking process up to the point of consumption. Therefore, it is important to acquire information on the persistence and germination of *C. difficile* spores in cooked food and to define the infectious dose for this bacterium.

Our results support the idea that young animals colonized by *C. difficile* may represent an important source of *C. difficile* strains, often being resistant to antibiotics, and highlight the importance of efficient surveillance and prevention programs against CDI in these farm animals.

Finally, the rapid emergence at the local and global levels of new *C. difficile* types of interest for veterinary and human medicine, such as the emergent RT 569 detected in this study, requires integrated collaboration among public health authorities, veterinary medicine and agriculture in order to control and prevent infections that are able to cross species and geographical barriers.

## Figures and Tables

**Table 1:** Types of animal samples analyzed and numbers of *C. difficile* strains and PCR ribotypes detected in the different investigations carried out in this study.

Investigation	Animal Species (Type of Sample)	Age Class (Clinical Data)	N. Samples Tested	N. Positive (%)	Ribotypes (N. of Strains)
***Passive surveillance on neonatal animals***	Swine (feces/intestinal content)	suckling piglet in the first 2 weeks of life (live animals with diarrhea or animals that had died with enterocolitis)	493	143 (29.0%)	078 (130), 620 (4), 066/2 (3), 068 (2), 126 (2), 085 (1), 569 (1)
	Bovine (feces/intestinal content)	calves in the first 3 weeks of life (live animals with diarrhea or animals that had died with enterocolitis)	140	2 (1.4%)	033 (1), 078 (1)
***Cross-sectional study at farms***	Swine (feces)	suckling piglets in the first 2 weeks of life (with diarrhea)	179	39 (21.8%)	078 (32), 126 (3), 569 (2), 620 (2)
		suckling piglets in the first 2 weeks of life (without diarrhea)	271	51 (18.8%)	078 (41), 620 (5), 126 (3), 068 (1), 569 (1)
		weaned piglets	45	2 (4.4%)	078 (2)
		grower pigs	45	0 (0.0%)	
		finisher pigs	90	1 (1.1%)	001 (1)
		breeding sows	90	12 (13.3%)	078 (12)
	Bovine (feces)	calves in the first 3 weeks of life (with diarrhea)	51	3 (5.9%)	033 (2), 078 (1)
		calves in the first 3 weeks of life (without diarrhea)	99	7 (7.1%)	126 (3), 033 (2), 078 (1), 045/2 (1)
		2–3-month-old heifers	30	0 (0.0%)	
		4–5-month-old heifers	30	1 (3.3%)	078 (1)
		6–12-month-old heifers	30	1 (3.3%)	033 (1)
		13–18-month-old heifers	30	0 (0.0%)	
		19–24-month-old heifers	30	1 (3.3%)	033 (1)
		primiparous cows	30	0 (0.0%)	
		pluriparous cows	30	0 (0.0%)	
***Cross-sectional study at slaughterhouses***	Swine (intestinal content)	Italian heavy pig (160–180 Kg b.w.)	113	2 (1.8%)	078 (1), 005 (1)
	Swine (carcass swab)	Italian heavy pig (160–180 Kg b.w.)	113	0 (0.0%)	
	Bovine (intestinal content)	dairy cows	100	0 (0.0%)	
	Bovine (carcass swab)	dairy cows	100	2 (2.0%)	126 (1) PR23597 (1)
		**Tot.**	**2139**	**267 (12.5%)**	

**Table 2:** Characteristics of CDI in patients and typing of the human *C. difficile* strains analyzed in this study.

Onset CDI ^§^ (N. of Strains)	Age of Patients	Gender of Patients * (N. of Strains)	PCR-Ribotype	N. of Strains
HA-CDI (61)	62	M (1)	001	1
	78	F (1)	002	1
	76–89	M (2), F (2)	005	4
	87	F (1)	012	1
	35–99	M (4), F (1)	014	5
	65–92	M (6), F (19)	018	25
	59–97	M (3), F (5)	078	8
	76–89	M (2)	085	2
	35–82	M (1), F (1)	126	2
	54	M (1)	220	1
	84	F (1)	241	1
	68	M (1)	427	1
	79	F (1)	446	1
	81	F (1)	449	1
	53–79	F (2)	607	2
	92	M (1)	620	1
	60	F (1)	743	1
	81	F (1)	AI-82/1	1
	80–81	M (2)	PR18626	2
CA-CDI (25)	65–81	F (3)	002	3
	74	F (1)	003	1
	78	M (1)	005	1
	96	F (1)	014	1
	51–91	M (2), F (5)	018	7
	84–86	F (2)	020	2
	81	F (2)	054	2
	1	F (1)	087	1
	1	M (1)	106	1
	54	M (1)	220	1
	42–85	F (2)	425	2
	79–86	M (2)	569	2
	95	F (1)	607	1
LTCFs (11)	93–95	F (2)	014	2
	77–95	M (1), F (5)	018	6
	97	F (1)	023	1
	92	M (1)	033/1	1
	87	F (1)	126	1

^§^ HA-CDI: hospital-acquired CDI; CA-CDI: community-acquired CDI; LTCFs: long-term care facilities; * F: female; M: male.

**Table 3:** Molecular characterization of the animal and human *C. difficile* strains investigated in this study.

Origin	N. of Strains (Species)	Toxin Genes Profile *	PCR-Ribotypes (N. of Strains)
**Animal**	252 (243 porcine 9 bovine)	*tcdA+*/*tcdB+*/*cdtA+*/*cdtB+*	045 (1), 066/2 (3), 078 (225), 126 (12), 620 (11)
	7 (bovine)	*tcdA+*/*tcdB*−/*cdtA+*/*cdtB+*	033 (7)
	6 (porcine)	*tcdA+*/*tcdB+*/*cdtA*−/*cdtB*−	001 (1), 005 (1), 569 (4)
	2 (1 porcine 1 bovine)	*tcdA*−/*tcdB*−/*cdtA*−/*cdtB*−	085 (1), PR23597 (1)
**Human**	16	*tcdA+*/*tcdB+*/*cdtA+*/*cdtB+*	023 (1), 033/1 (1), 078 (8), 126 (3), 427 (1), 620 (1), 743 (1)
	79	*tcdA+*/*tcdB+*/*cdtA*−/*cdtB*−	001 (1), 002 (4), 003 (1), 005 (5), 012 (1), 014 (8), 018 (38), 020 (2), 054 (2), 087 (1), 106 (1), 220 (2), 241 (1), 425 (2), 446 (1), 449 (1), 569 (2), 607 (3), AI-82/1 (1), PR18626 (2)
	2	*tcdA*−/*tcdB*−/*cdtA*−/*cdtB*−	085 (2)

* The *tcdA* gene encodes for toxin A; the *tcdB* gene encodes for toxin B; the *cdtA* and the *cdtB* genes encode for the binary toxin CDT subunits. Note: + PCR positive; − PCR negative.

**Table 4:** Distribution of the *C. difficile* RTs in the animal samples investigated in this study and their presence in human samples.

*C. difficile* RT	Animal Species (N. of Strains and Status)	Human CDI Onset * (N. of Strains)
001	Swine (1 asymptomatic)	HA-CDI (1)
005	Swine (1 asymptomatic)	CA-CDI (1) HA-CDI (4)
033	Cattle (3 symptomatic, 4 asymptomatic)	-
045	Cattle (1 asymptomatic)	-
066/2	Swine (3 symptomatic)	-
078	Swine (165 symptomatic, 56 asymptomatic)	HA-CDI (8)
	Cattle (2 symptomatic, 2 asymptomatic)	
085	Swine (1 symptomatic)	HA-CDI (2)
126	Swine (8 symptomatic)	HA-CDI (2) LTCF (1)
	Cattle (4 asymptomatic)	
569	Swine (3 symptomatic, 1 asymptomatic)	CA-CDI (2)
620	Swine (6 symptomatic, 5 asymptomatic)	HA-CDI (1)
PR23597	Cattle (1 Asymptomatic)	-

* HA-CDI: hospital acquired CDI; CA-CDI: community-acquired CDI; LTCF: long-term care facility.

**Table 5:** Antibiotic susceptibility of the animal and human *C. difficile* strains investigated in this study.

Erythromycin (Breakpoint: 8 mg/L)					
**Origin**	MIC Range (µg/mL)	MIC 90 (µg/mL)	MIC 50 (µg/mL)	N. of Resistant Strains (%)	Ribotypes (N. of Strains)
Human	≤0.016–≥256	≥256	≥256	59 (62%)	012 (1) 014 (1) 018 (38) 078 (7) 085 (2) 126 (3) 220 (2) 569 (2) 607 (2) 620 (1)
Porcine	0.25–≥256	≥256	≥256	130 (93%)	078 (113) 085 (1) 126 (4) 569 (4) 620 (8)
Bovine	0.125–≥256	≥256	0.25	7 (47%)	078 (4) 126 (3)
**Moxifloxacin (Breakpoint: 8 mg/L)**					
**Origin**	**MIC range** (µg/mL)	**MIC 90** (µg/mL)	**MIC 50** (µg/mL)	**N. of resistant strains (%)**	**Ribotypes (n. of strains)**
Human	0.38–≥32	≥32	4	46 (48%)	012 (1) 014 (1) 018 (37) 078 (3) 126 (2) 607 (2)
Porcine	0.25–≥32	≥32	8	71 (52%)	066/2 (3) 078 (57) 085 (1) 126 (3) 620 (7)
Bovine	0.25–≥32	≥32	0.5	2 (13%)	045 (1) 078 (1)
**Tetracycline (Breakpoint: 16 mg/L)**					
**Origin**	**MIC range** (µg/mL)	**MIC 90** (µg/mL)	**MIC 50** (µg/mL)	**N. of resistant strains (%)**	**Ribotypes (n. of strains)**
Human	≤0.016–16	3	0.047	1 (1%)	220 (1)
Porcine	0.023–12	6	3	0	-
Bovine	0.032–8	4	0.064	0	-
**Metronidazole (Breakpoint: 2 mg/L)**					
**Origin**	**MIC range** (µg/mL)	**MIC 90** (µg/mL)	**MIC 50** (µg/mL)	**N. of resistant strains (%)**	**Ribotypes (n. of strains)**
Human	≤0.016–0.32	0.094	0.047	0	-
Porcine	≤0.016–0.125	0.094	0.047	0	-
Bovine	≤0.016–0.19	0.125	0.064	0	-
**Amoxycillin (Breakpoint: 16 mg/L)**					
**Origin**	**MIC range** (µg/mL)	**MIC 90** (µg/mL)	**MIC 50** (µg/mL)	**N. of resistant strains (%)**	**Ribotypes (n. of strains)**
Human	0.064–4	1	0.038	0	-
Porcine	0.094–0.047	0.38	0.25	0	-
Bovine	0.125–0.5	0.5	0.125	0	-
**Vancomycin (Breakpoint: 2 mg/L)**					
**Origin**	**MIC range** (µg/mL)	**MIC 90** (µg/mL)	**MIC 50** (µg/mL)	**N. of resistant strains (%)**	**Ribotypes (n. of strains)**
Human	0.75–2	2	1.5	0	-
Porcine	0.5–1.5	1.5	1.5	0	-
Bovine	0.75–1.5	1.5	1.5	0	-

**Table 6:** Antibiotic resistance mechanisms found in the *C. difficile* strains isolated from animals in this study.

Origin	Antibiotic Resistance Molecular Profile ^a^	PCR-Ribotypes (N. of Strains) ^c^
**Porcine**	Thr82-Ile (25)	**066/2 (2),** 078 (23), **126 (1)**
	Thr82-Ile + *ermB* (6)	078 (5)
	Thr82-Ile + *ermB* + *tetM* + *tetO* (5)	078 (4), **126 (1)**
	Thr82-Ile + *ermB* + *tetM* + *tetW* (2)	078 (2)
	Thr82-Ile + *ermB* + *tetM* + *tetO* + *tetW* (2)	078 (2)
	Thr82-Ile + *tetM* (9)	066/2 (1), 078 (7), 620 (1)
	Thr82-Ile + *tetM* + *tetO* (18)	078 (13), **620 (5)**
	Thr82-Ile + *tetM* + *tetO* + *tetW* (3)	**126 (2), 620 (1)**
	Thr82-Ile + *ermQ* (1)	078 (1)
	Thr82-Val + *tetM* (1)	**085 (1)**
	*ermB* (9)	078 (9)
	*ermB* + *tetM* + *tetO* (2)	078 (2)
	*ermB* + *tetM* + *tetO* + *tetW* (2)	078 (2)
	*tetM* (1)	078 (1)
	*tetM* + *tetO* (11)	078 (10), **620 (1)**
	*tetO* (1)	**569 (1)**
	No resistance genes (37) ^b^	078 (34), **569 (3)**
**Bovine**	Thr82-Ile (2)	**045 (1)**, 078 (1)
	*ermB* (1)	**126 (1)**
	*ermB* + *tetM* + *tetO* (1)	**126 (1)**
	*ermB* + *tetM* + *tetO* + *tetW* (1)	**126 (1)**
	*tetM* + *tetO* + *tetW* (1)	078 (1)
	No resistance genes (2) ^b^	078 (2)

^a^ All *C. difficile* strains with MICs for tetracycline between 4 and 12 mg/L^−1^ were analyzed for the presence of *tet* genes. ^b^ *C. difficile* strains resistant to ERY but negative for *erm* genes tested. ^c^ RTs different from RT 078 are in bold.

**Table 7:** Antibiotic resistance mechanisms found in the human *C. difficile* strains investigated in this study.

Onset (N. of Strains)	Mechanisms of Resistance (N. of Strains) *	PCR-Ribotypes (N. of Strains)
HA-CDI (43)	Thr82Ile (24)	002 (1), 014 (1), 018 (19), 078 (2), 607 (1)
	Thr82Ile + *ermB* (5)	018 (4), 126 (1)
	Thr82Ile + *ermB* + *tetM* (1)	078 (1)
	Thr82-Ile + *ermQ* (2)	018 (2)
	*ermB* (4)	012 (1), 078 (1), 085 (2)
	*ermB* + *tetM* (2)	078 (1), 220 (1)
	*tetM* + *tetO* (3)	078 (2), 126 (1)
	No substitutions nor resistance genes (2) ^§^	078 (1), 620 (1)
CA-CDI (11)	Thr82Ile (8)	018 (7), 607 (1)
	*ermB* + *tetM* (1)	220 (1)
	*ermQ* (1)	569 (1)
	No substitutions nor resistance genes (1) ^§^	569 (1)
LTCF (7)	Thr82Ile (6)	018 (5), 126 (1)
	Thr82-Ile + *ermC* + *tetM* + *tetW* (1)	018 (1)

* All *C. difficile* strains with MICs for tetracycline between 4 and 12 mg/L were analyzed for the presence of *tet* genes. ^§^ *C. difficile* strains negative for *erm* genes were resistant to erythromycin.

## Data Availability

Not applicable.
